# Tyramine Actions on *Drosophila* Flight Behavior Are Affected by a Glial Dehydrogenase/Reductase

**DOI:** 10.3389/fnsys.2017.00068

**Published:** 2017-09-27

**Authors:** Stefanie Ryglewski, Carsten Duch, Benjamin Altenhein

**Affiliations:** ^1^Institute of Developmental Biology and Neurobiology, Johannes Gutenberg-Universität Mainz, Mainz, Germany; ^2^Institute of Zoology, University of Cologne, Cologne, Germany

**Keywords:** *Drosophila*, biogenic amine, tyramine, flight, modulation, glia

## Abstract

The biogenic amines octopamine (OA) and tyramine (TA) modulate insect motor behavior in an antagonistic manner. OA generally enhances locomotor behaviors such as *Drosophila* larval crawling and flight, whereas TA decreases locomotor activity. However, the mechanisms and cellular targets of TA modulation of locomotor activity are incompletely understood. This study combines immunocytochemistry, genetics and flight behavioral assays in the *Drosophila* model system to test the role of a candidate enzyme for TA catabolism, named Nazgul (Naz), in flight motor behavioral control. We hypothesize that the dehydrogenase/reductase Naz represents a critical step in TA catabolism. Immunocytochemistry reveals that Naz is localized to a subset of Repo positive glial cells with cell bodies along the motor neuropil borders and numerous positive Naz arborizations extending into the synaptic flight motor neuropil. RNAi knock down of Naz in Repo positive glial cells reduces Naz protein level below detection level by Western blotting. The resulting consequence is a reduction in flight durations, thus mimicking known motor behavioral phenotypes as resulting from increased TA levels. In accord with the interpretation that reduced TA degradation by Naz results in increased TA levels in the flight motor neuropil, the motor behavioral phenotype can be rescued by blocking TA receptors. Our findings indicate that TA modulates flight motor behavior by acting on central circuitry and that TA is normally taken up from the central motor neuropil by Repo-positive glial cells, desaminated and further degraded by Naz.

## Introduction

Neuromodulatory substances shape central pattern generator (CPG) network and motoneuronal (MN) activity into many different forms, thus lending flexibility of the motor output to different behavioral requirements or to different internal states (Harris-Warrick and Marder, 1991). Modulators can act on many different levels of the motor system, ranging from brain circuitry and the motor-network, to actions on sensory neurons, neuromuscular transmission and muscle properties. In many cases, several different modulators affect motor output, and vice versa, the same modulator may act on multiple different levels (Marder et al., 2005).

The highest level of modulatory monoamine input occurs during “fight or flight” behavioral situations (Cannon, 1932). Analogous to noradrenaline (NA) in vertebrates, in insects “fight or flight” reactions are often attributed to the biogenic amine octopamine (OA; Stevenson and Rillich, 2012). Both *Drosophila* larval crawling (Fox et al., 2006) and adult flight motor behaviors (Brembs et al., 2007) are facilitated by OA. Moreover, in invertebrates the biogenic amine tyramine (TA) regulates motor behaviors in an antagonistic manner to OA (Pflüger and Duch, 2011). Larval *Drosophila* crawling (Saraswati et al., 2004; Fox et al., 2006) and adult flight (Brembs et al., 2007) initiation and maintenance are augmented by OA but reduced by TA signaling.

However, for both OA and TA the cellular site(s) of action that underlie the modulation of motor behavior remain largely unknown. With regard to insect flight the biogenic amine OA has been reported to affect central pattern generating circuits (Sombati and Hoyle, 1984), sensory sensitivity (Büschges et al., 1993; Ramirez et al., 1993; Matheson, 1997), flight muscle contraction properties, hormone release (Orchard et al., 1993) and muscle metabolism (Mentel et al., 2003). Although the effects of TA are less well described, TA is known to act also on insect central pattern generating circuits (Rillich et al., 2013), muscle contraction properties (Ormerod et al., 2013), metabolism (Downer, 1979) and likely also on sensory systems (Kutsukake et al., 2000). Accordingly, it is difficult to pinpoint whether peripheral or central actions of OA and TA mediate their known effects on flight behavior. Ideally, OA and TA actions have to be selectively manipulated at either site of action to test the resulting effects on motor behavior separately.

In neurons, TA is synthesized from the amino acid tyrosine by the enzyme tyrosine decarboxylase (TDC2, Roeder, 2005). TA can then be further processed into OA by the tyramine beta hydroxylase (Tβh, Monastirioti et al., 1996). Therefore, insect octopaminergic neurons also contain TA. Accordingly *Drosophila* *tβh* null mutants lack OA but have strongly increased TA levels (Monastirioti et al., 1996). The results are reduced flight durations and fewer flight initiations, and these behavioral phenotypes can be partially rescued by feeding OA or by blocking TA receptors, thus demonstrating that both amines affect flight motor behavior in an antagonistic manner (Brembs et al., 2007).

This study aims to providing some insight as to whether TA affects flight motor behavior by modulatory actions in the central nervous system (CNS). In an effort to selectively manipulate TA levels in the CNS we employ the genetic power of *Drosophila* to interfere with the putative degradation pathway of TA in glial cells. In general, monoamines are degraded by desamination by monoamine oxidases (MAOs) to aldehydes, which are further processed by dehydrogenases/reductases. Recently, in *Drosophila* a respective candidate dehydrogenase/reductase (accession number CG31235) for TA degradation has been identified and named Nazgul (Naz; de Visser, 2016).

We find that Naz localizes to a specific set of glial cells in the CNS. Furthermore, targeted RNAi knock down of *nazgul* in glial cells phenocopies decreased flight durations as induced by increased TA levels. These data indicate that Nazgul indeed takes place in TA degradation and that TA modulates flight motor behavior at least in part by modulatory actions in the CNS.

## Materials and Methods

### Animals

*Drosophila melanogaster* were reared in standard 68 ml plastic vials with foam stoppers on a yeast-cornmeal-syrup-agar diet at 25°C and 55% humidity with a 12-h light/dark regimen. Fly food contained the following ingredients (per 1000 ml): 116 g glucose (Carl Roth, Germany, HN06.04), 55 g cornmeal (Rapunzel, Germany, Demeter standard 420505), 11 g agar (Roth 5210.5), 29 g active dry yeast (Huber Mühle, Germany), 0.6 g ascorbic acid (Roth 3525.3) and 12.2 ml of 10% tegosept (Apex BioResearch Products 20–258) in 100% ethanol. All experiments were carried out with 2–3 days old adult male flies. To drive expression of UAS-transgenes in glial cells we used *repo-GAL4* (w^1118^; P{GAL4}repo/TM3, Sb^1^; Bloomington # 7415). To knock down Naz we used a UAS-*naz*-RNAi flystrain (VDRC RNAi fly stock center # 107974). To enhance RNAi knock down efficacy we included an extra copy of dicer 2 (UAS-Dcr2, Bloomington Stock 24650, Dietzl et al., 2007). To drive expression of UAS transgenes in muscle cells we used Mef2a-GAL4 (Myocyte enhancer factor 2; y w[*]; P{w[+mC] = GAL4-Mef2.R}3; Bloomington # 27390).

### Behavioral Testing

Two to three days old male flies were immobilized by cooling for 30 s in an empty 68 ml plastic vial on ice and then immediately transferred onto a cold plate at 2°C. Then animals were glued (clear glass adhesive (Duro; Pacer Technology, Rancho Cucamonga, CA, USA)) with head and thorax to a triangle-shaped copper hook (0.02 mm diameter). Adhesion was achieved by exposure to UV light for 10 s. The animals were then immediately returned to room temperature and kept individually in small chambers containing a few grains of sucrose on filter paper until testing (2–3 h). Flies, glued to copper hooks were attached to the experimental setup via a clamp to accomplish stationary flight. For observation, the fly was illuminated from behind and above 150 W (15 V; Schott, Elmsford, NY, USA) and fixed in front of a paper panel with horizontal white and black stripe patterns. Tarsal contact with a bead of polystyrene prevented flight initiation before the experiment started. For flight initiation, the polystyrene bead was removed, and the fly was gently aspirated by mouth to produce an air current of about 0.8 m per second. The time until the fly first stopped flying was measured. After each flight bout the fly was again gently aspirated as a stimulation to fly. The time was recorded for each flight bout. The experiment was completed when no flight reaction was initiated in response to three consecutive air stimuli. The person recording the flight times was unaware of the genotype of the animal.

### Western Blotting

Brains of 2–3 day-old male adult *Drosophila* were dissected according to Wu and Luo (2006). For SDS-PAGE Western blot 10 brains per lane (40 ml Hoefer Western blot chamber with 1.5 mm spacer and comb with 15 × 100 μl pockets) were dissected and mechanically homogenized with a clean micro-pestle in 85 μl ice cold 2× concentrated sample buffer (7.5 ml 4× Tris-HCl/SDS, pH 6.8, 6 ml glycerol, 1.5 g SDS, 0.3 g dithiothreitol (DTT), ~1 mg bromophenol blue in 30 ml with ddH_2_O). 4× Tris-HCl was prepared with 40 ml ddH_2_O, 6.05 g Tris base, 0.4 g SDS, pH was adjusted to 6.8 with HCl, then filled up to 100 ml with ddH_2_O and filtered through a 0.45 μm sterile filter. Homogenized samples were boiled for 3 min and then loaded into the gel chambers. We used a 5% polyacrylamide stacking gel (10 ml, 1.7 ml of 30% bis-acrylamide, 1.25 ml 4× Tris/SDS pH 8.8, 6.8 ml ddH_2_O, 100 μl 10% ammoniumpersulfate (APS), 10 μl TEMED) and let the samples run through a 10% polyacrylamide running gel (30 ml, 13.3 ml 30% bis-acrylamide, 10 ml 4× Tris/SDS pH 8.8, 16 ml ddH_2_O, 400 μl 10% APS, 16 μl TEMED. APS and TEMED were added just prior to pouring the gel. Samples were run through the stacking gel at 20 mA and then through the running gel at 30 mA constant current at room temperature. As protein marker 20 μl Precision Plus Protein^™^ WesternC^™^ Blotting Standard 10–250 kDa was used (Bio Rad #161-0376). Proteins were then transferred onto nitrocellulose membrane (Bio Rad) overnight at 4°C at 35 V constant voltage in a 5 L 42E Hoefer blotting chamber using transfer buffer (18.2 g Tris base, 86.5 g glycine, 900 ml methanol, fill up to 6 L with ddH_2_O). Nitrocellulose membrane was incubated in blocking solution (10% milk powder in electrophoresis buffer with Tween-20 (TBST) for 2 h at room temperature. Before antibody incubation, nitrocellulose membrane was cut horizontally because both primary antibodies were made in rabbit. Primary antibodies were prepared in 2.5% milk powder in TBST and the nitrocellulose membrane incubated overnight: rabbit α-hsp90 (New England Biolabs or Cell Signaling Technology, #4872S) as loading control 1:1000 and rabbit α-Naz 1:250 (von Hilchen et al., 2013) were used. Secondary antibody incubation was done together for both pieces of the nitrocellulose membrane. Secondary antibody was applied for 2 h at RT: goat α-rabbit (H + L) HRP conjugate 1:5000 (Millipore #AP106P) was used. Protein bands were detected with luminol-based Immobilon Western Chemiluminescence detection kit (Millipore #WBKLS01 00). Hsp90 band was expected at 84 kDa, Naz (CG31235) was expected at 46 kDa. Pictures were taken and saved as .tiff image files.

### Immunocytochemistry

Adult flies were dissected in standard saline along the dorsal midline and the gut was removed to expose the ventral nerve cord (VNC) as described previously (Boerner and Duch, 2010). Next preparations were fixed for 60 min in 4% PFA in 0.1 M PBS, washed 6 × 20 min 0.1 M PBS and 6 × 30 min in PBS Triton-X 0.5%. This was followed by primary antibody incubation overnight at 4°C in 0.1 m PBS with 0.2% BSA and 0.3% Triton-X on a shaker. Rabbit α-Naz and guinea pig α-Repo (von Hilchen et al., 2013) were both used at 1:1000 in 0.1 M PBS Triton-X 0.1%. Specificity for both primary antibodies has previously been reported (von Hilchen et al., 2013). Specificity for α-Naz has been further confirmed in this study by the absence of immunopositive signal in Western blots after *Naz*-RNAi knock down.

Following primary AB incubation preparations were rinsed in 0.1 M PBS and washed in 0.1 M PBS 6 × 30 min. For detection of Naz immunolabel donkey α-rabbit secondary antibody coupled to Alexa 568 (Invitrogen A10042) was used. For detection of Repo immunolabel goat α-guinea pig secondary antibody coupled to Cy5 (Dianova 106-605-003) was used. Both secondary antibodies were incubated at a concentration of 1:500 in 0.1 M PBS overnight at 4°C on a shaker. Next preparations were rinsed for 6 × 30 min with PBS followed by an ascending ethanol series (50, 70, 90, 100%), and then mounted in methylsalicylate. Immunolabel was visualized using a Leica SP8 confocal laser-scanning microscope (Leica, Germany). Alexa 568 was excited with a 568 solid state laser and detection was set between 580 nm and 610 nm. Cy5 was excited with a 633 Helium Neon laser and detection was set between 640 nm and 690 nm. Images were processed with Amira 5.3.3 (Mercury Systems) and Corel X7 (Corel Corporation) software.

Thoracic neuromeres, lateral nerves and neuropil borders were labeled and defined as previously described (Boerner and Duch, 2010). The thoracic neuropils are characterized by the absence of any cell bodies and a glial lining that separates nervous tissue densely packed with synapses from a VNC cortex packed with cell bodies. The demarcation line can be visualized in single optical sections through the VNC.

### Statistical Analysis

All flight behavioral data are presented as box blots with median, 25, and 75 percentiles. Error bars represent the 10 and the 90 percentiles. Statistical differences were tested for with Kruskal Wallis ANOVA. Following a statistical significant Kruskal-Wallis *p* value (significance level was set <0.05) Dunn-Bonferroni *post hoc* method was used for between groups *post hoc* comparisons. For between groups comparisons * indicates *p* < 0.05 and ** indicates *p* < 0.01, whereas ns indicates *p* > 0.1. All statistical testing was conducted with SPSS Statistics 22 software.

## Results

**Figure 1:**
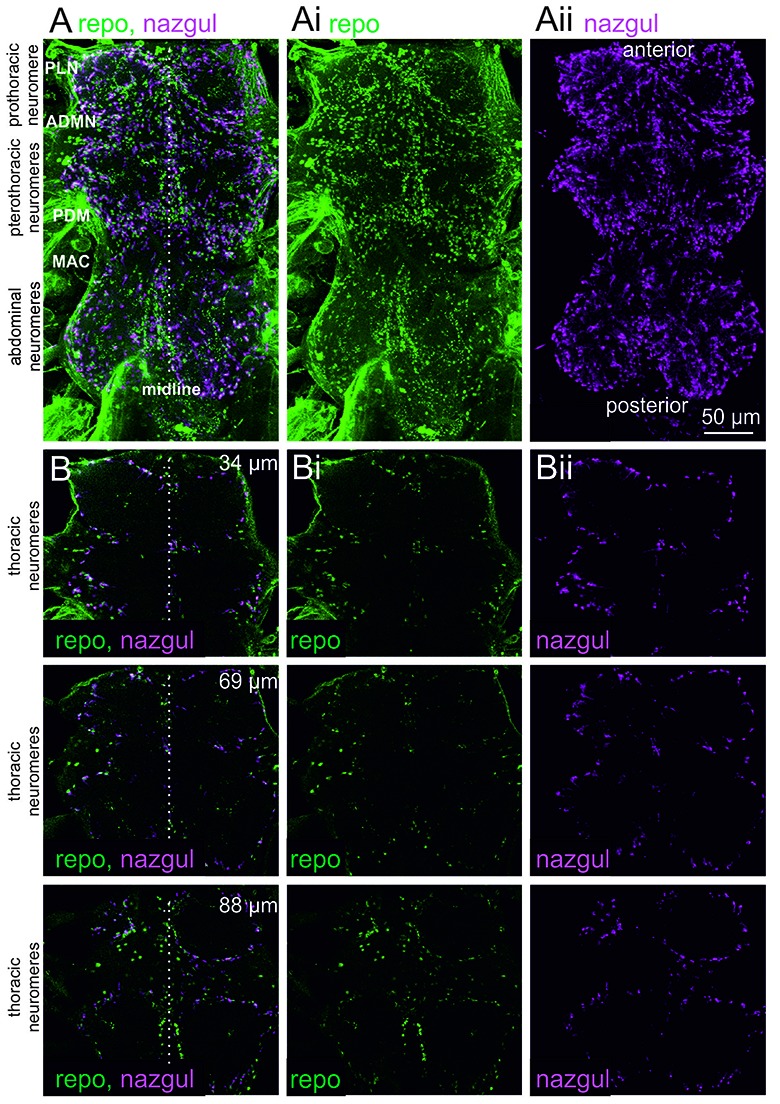
Nazgul (Naz) localizes in similar patterns as Repo positive glial cells in the ventral nerve cord (VNC). **(A)** Maximum projection views of a representative confocal image stack taken from double immunolabeling of Naz (magenta) and Repo (green) in the VNC. The locations of the prothoracic, pterothoracic (meso- and metathoracic) and abdominal neuromeres are indicated. The midline is indicated by a dotted white line and prominent nerves are labeled (PLN, prothoracic leg nerve; ADMN, anterior dorsal mesothoracic nerve; PDM, posterior dorsal mesothoracic nerve; MAC, mesothoracic accessory nerve). The individual labels for Repo and Naz are shown in **(Ai,Aii)**. **(B)** Single optical sections of Naz (magenta) and Repo (green) label at 34 μm (upper row), 69 μm (middle row) and 88 μm (lower row) imaging depth show that Naz and Repo positive cells localize to the borders of the motor neuropils. The individual labels for Repo and Naz are shown in **(Bi,Bii)**.

We first analyzed the localization of the dehydrogenase/reductase, Naz, in the adult *Drosophila* VNC by immunohistochemistry and confocal laser scanning microscopy (Figure 1). Maximum projection views from optical sections through the entire VNC revealed many Naz positive cells in all three throracic and all abdominal neuromeres (Figure 1A, magenta). The nuclei of glial cells in the CNS were co-labeled with the glial cell marker Reversed polarity (Figure 1A, green), Repo, which is required for glial cell differentiation (Halter et al., 1995). This indicated that Naz was localized to glial cells. Single optical sections (Figure 1B) at different depths (34, 69 and 88 μm) indicated that Naz was predominantly localized to glial cells which demarked the borders of the neuropil regions.

**Figure 2:**
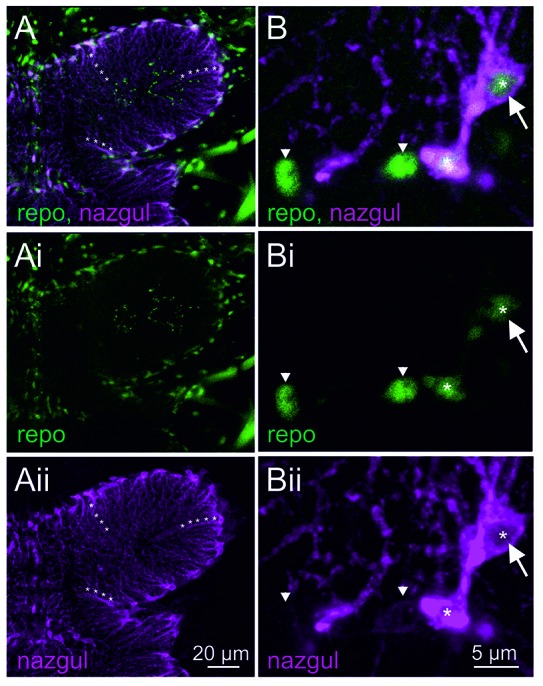
Naz localizes to the cytosol of a subset of Repo positive glial cells. **(A)** Maximum projection view of a representative confocal image stack taken from double immunolabeling of Naz (magenta) and Repo (green) thoracic flight motor neuropil. Lines of white asterisks demark arbors of Naz positive cells that extend from the neuropil border into the center of the neuropil. The individual labels for Repo and Naz are shown in **(Ai,Aii)**. **(B)** Selective enlargement of a representative single optical section shows four Repo positive glial cell nuclei, two belonging to Naz negative (arrowheads) and two to Naz positive glial cells. Only a subset of Repo positive glial cells is Naz positive. Naz protein is localized cytosolic through the soma and the arbors of these glial cells. Naz protein is not localized to the nucleus as apparent from the absence of Naz immunopositive label in single optical sections through Repo positive nuclei (white arrow). Please note that the lower Repo-positive glial cell nucleus is out of focus in this section, and thus co-labels with Naz-positive cytosol. The individual labels for Repo and Naz are shown in **(Bi,Bii)**.

Selective enlargement of the mesothoracic neuropil showed Repo positive glial cells around the neuropil border (Figures 2A,Ai, green). Many of the Repo positive glial cells were also Naz positive (Figures 2A,Aii, magenta). Similarly, Naz has previously been used as a marker for longitudinal glial cells during embryonic development (von Hilchen et al., 2010). Please note that Repo labeled the nuclei of these glia cells, but by contrast, Naz was localized cytosolically and labeled the soma and glial cell processes that extended into the flight motor neuropil (as examples three such processes are labeled with white asterisks in Figures 2A,Aii). Careful inspection of image stacks from five animals revealed that every Naz positive cell in the VNC was also Repo positive. Representative selective enlargements of few cells revealed two things: first, Naz was always localized to the cytosol but not to the Repo positive nucleus of the glial cells (Figure 2B white arrow, single optical section through the glial cell nucleus). Second, all Naz positive cells contained also a Repo positive nucleus, but not all Repo positive glial cells were also Naz positive (Figure 2B). For visualization, in Figures 2B,Bi,Bii Naz negative glial cells are labeled by white arrowheads, whereas Naz positive glial cells are labeled by white asterisks. Therefore, Naz localized to a subset of glial cells that align the flight motor neuropil borders and project extensions into the central neuropil regions.

**Figure 3:**
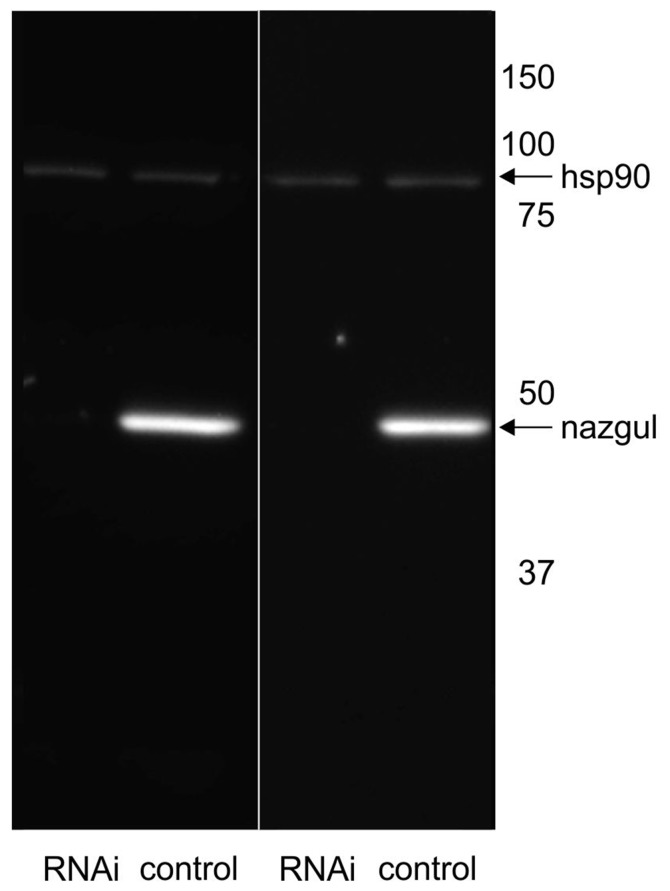
Naz-RNAi effectively knocks down Naz protein. Western blotting with control fly homogenate reveals a prominent band at the predicted size of the Naz protein (45.5 kD) that is not detectable in homogenate from flies with expression of UAS-*naz*-RNAi under the control of Repo-GAL4. Heat shock protein 90 (hsp90) serves as loading control and is detected at similar levels in control and *naz-*RNAi knock down animals.

We next tested whether Naz expression could be eliminated by targeted RNAi knock down in Repo positive glial cells under the control of *repo*-GAL4. RNAi knock down efficacy was enhanced by inclusion of extra Dicer-2 (UAS-Dcr2, Bloomington Stock 24650, Dietzl et al., 2007). We have previously reported that this effectively enhances knock down strength (Ryglewski et al., 2012; Hutchinson et al., 2014). As controls we crossed UAS-Dcr2 to *repo*-GAL4 but did not include UAS-*naz*-RNAi. In controls Western blotting revealed one prominent band at the predicted size for the Naz protein at 45.5 kD (Figure 3). By contrast, following targeted RNAi knock down VDRC (107974) of *naz* in *repo* expressing glial cells no Naz protein was detectable by Western blotting (Figure 3). Therefore, RNAi effectively knocked down Naz protein below detection threshold. This confirmed that Naz was exclusively expressed in *repo* positive glial cells because RNAi knock down was targeted selectively to glial cells. It also further confirmed Naz antibody specificity because antibody detection was eliminated by protein knock down with an RNAi that has no reported off-target effects. Given that we found no Naz immunopositive signal in the periphery and that Repo positive glial cells are located in the adult CNS we next utilized *naz* RNAi knock down to test possible effects on flight motor behavior (see “Materials and Methods” Section for flight behavioral assay).

**Figure 4:**
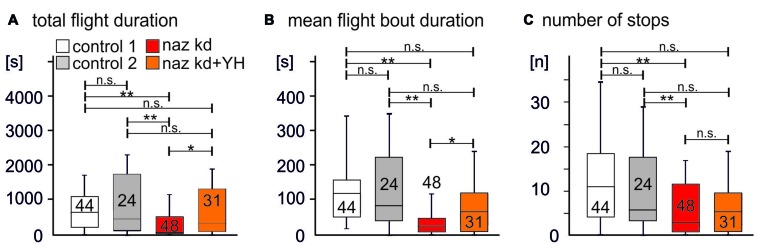
Knock down of Naz reduces flight durations. **(A)** Total flight duration is significantly reduced in animals with knock down of Naz in Repo positive glial cells (Repo-GAL4 × UAS-*naz*-RNAi; UAS-Drc2, red bar; naz kd) as separately compared to two different controls (control 1, white bar; expression of UAS-drc2 under the control of Repo-GAL4; control 2, gray bar; expression of UAS-drc2 and UAS-*naz*-RNAi under the control of Mef2A). Total flight duration of animals with knock down of Naz in Repo positive glial cells is significantly increased by feeding of the TAR blocker Yohimbine (YH; naz kd + YH, orange bar). Feeding YH to animals with naz-RNAi knock down yields flight durations that are not significantly different from those of control 1 or of control 2. **(B)** Similarly, mean flight bout duration is significantly reduced in animals with knock down of Naz in Repo positive glial cells as compared to each control, and this phenotype if also rescued by feeding of YH. **(C)** The number of flight bouts is also significantly reduced in animals with knock down of Naz in Repo positive glial cells as compared to each control. Feeding of YH increase the mean of flight bouts slightly but not significantly. Kruskal Wallis ANOVA with Dunn’s *post hoc* testing, **p < 0.01, *p < 0.05, n.s. *p* > 0.1.

Flies with *naz* RNAi knock down in glial cells (Figure 4A, red bar) showed significantly shorter total flight durations as compared to two different controls (Figure 4A, white and gray bars, *p* = 0.0004 for control 1 vs. Naz-RNAi, *p* = 0.0027 for control 2 vs. *naz*-RNAi). As controls we used UAS-Dcr2 expressed under the control of *repo*-GAL4 (control 1, white bar) and expression of UAS-Dcr2 and UAS-*naz*-RNAi in muscle under the control of Mef2-GAL4 (control 2, gray bar). A control with RNAi expression in muscle was used to control for possible leak expression of the UAS-RNAi construct in the absence of GAL4. Given that *naz* knock down in the CNS reduced flight durations as previously reported for *tβh* mutant flies that lack OA but have increased TA levels (Brembs et al., 2007), we hypothesized that a reduction of Naz function caused increased TA levels in the flight motor neuropil. If this was correct, pharmacological blockade of TA signaling should provide a rescue. Feeding of the competitive α2 adrenergic receptor antagonist Yohimbine (YH) restored total flight durations to levels that were not statistically significantly different from either of the two controls (Figure 4, orange bar, *p* = 0.14 for comparison to control 1, *p* = 0.53 for comparison to control 2), but differed significantly from the *naz-RNAi* knock down group (*p* = 0.02). YH has been demonstrated to selectively block TA receptors in *Drosophila* (Arakawa et al., 1990; Saudou et al., 1990), and we have previously used it to rescue flight durations in *tβh* mutant flies (Brembs et al., 2007). Similarly to total flight durations the mean duration of individual flight bouts (Figure 4B) and the number of flight bouts (Figure 4C) were significantly reduced by RNAi knock down of *naz* (*p* < 0.01 for both separate comparisons of the RNAi group with each of the two control groups). Mean flight bout durations could also be rescued by feeding YH (Figure 4B, *P* > 0.1 for both separate comparisons of the YH rescue group with each of the two control groups, *p* = 0.018 for the comparison of the *naz-RNAi* group with the YH fed rescue group). The number of flight bouts was slightly but not significantly increased by feeding YH to *naz* knock down animals (*p* = 0.12).

## Discussion

### Naz Is Likely Involved in Reducing Biologically Active TA Levels in the Flight Motor Neuropil

Our data provide indirect evidence on the behavioral level that Naz normally functions to reduce biologically active TA levels in the flight motor neuropil, because knock down of Naz causes similar flight behavioral changes as observed with genetically increased TA levels (Brembs et al., 2007). Similarly to a reduction in flight durations in adult flies, increased TA levels reduce *Drosophila* larval crawling distances (Saraswati et al., 2004; Fox et al., 2006), and this phenotype is also recapitulated by knock down of Naz (de Visser, 2016). The hypothesis that Naz functions in the TA degradation pathway is supported by the finding that motor behavioral phenotypes as induced by *naz* knock down can be rescued by feeding the TAR blocker YH. Therefore, our data provide first hints to the mechanisms that might remove TA from the synaptic cleft. The most parsimonious explanation is that TA is taken up into Repo positive glial cells which are located at the neuropil borders and extend extensive processes into the flight motor neuropil. As typical for monoamines, TA is then likely desaminated by a MAO, and thus converted to p-hydroxyphenyl-acetaldehyde. Naz might then convert p-hydroxyphenyl-acetaldehyde further into p-hydroxyphenyl acetic acid (de Visser, 2016). We speculate that *naz* knock down causes accumulation of p-hydroxyphenyl-acetaldehyde, which in turn slows desamination of TA, and thus, transport of TA into glial cells. The resulting consequence would be higher extracellular TA levels in the flight motor neuropil. This scenario is consistent with our findings that *naz* knock down causes similar phenotypes as feeding TA or genetic upregulation of TA signaling (Brembs et al., 2007), and that pharmacological blockade TARs rescues the behavioral phenotype. This would mean that Naz function is rate limiting for TA uptake into glial cells. It would be instructive to test whether overexpression of Naz causes opposite effects on flight performance as compared to *naz*-RNAi, but we have so far not succeeded to produce a UAS-*naz* fly strain. However, it is widely accepted that biologically active monoamine levels in the synaptic cleft can be increased by blocking intracellular degradation. In Parkinson’s disease, for instance, pharmacological interventions with MAO function are utilized to enhance dopamine signaling (Unzeta and Sanz, 2011; Pathak et al., 2016). However, we have no direct evidence for the proposed function of Naz, and the transporter for TA into glial cells has also not yet been identified.

### TA Modulates Flight Motor Behavioral Likely by Actions in the CNS

It has long been known in multiple species that the biogenic amines OA and TA modulate insect motor behavior in an antagonistic manner (Roeder, 2005; Pflüger and Duch, 2011). In addition, numerous sites of OA and TA action have been identified, ranging from the modulation of central circuitry, neuromuscular transmission and muscle contraction properties, to sensory sensitivity, hormone release and muscle metabolism (Pflüger and Duch, 2011). However, at present the relative contributions of these different sites of action to the motor behavioral changes that are observed upon altered TA and/or OA signaling remain largely unknown. Our data indicate that central actions of TA might play a prominent role in the modulation of flight motor performance. We found prominent Naz localization in glial cells with arborizations in the central motor neuropils, but almost no Naz immunopositive cells in the periphery. Accordingly, knock down of Naz in glial cells restricts the manipulation mostly to the CNS. This has not been possible with genetic manipulations of the OA and TA synthesizing enzymes (*TDC2* and *Tβh*), the OA and TA receptors, or with drug feeding approaches. Given that we find significant reductions of flight durations and flight bout numbers, we suggest that the tyraminergic modulation of flight motor behavior is mediated to a large extent by central TA actions. In a next step it will be important to identify the cellular targets of TA action in the CNS. Given the localization of Naz positive glial arbors in the flight motor neuropil we suggest that premotor flight interneurons, flight motoneurons, or synapses between these cells might be promising targets for TA action. Accordingly, we have previously reported spatial overlap between the central arborizations of TDC2 positive OA/TA containing neurons and flight motoneuron dendrites in the VNC flight motor neuropil (Boerner and Duch, 2010). Alternative TA might act on brain circuitry that regulates the motivation to fly, but this is currently unknown. However, in vertebrate spinal cord, motoneurons are direct targets of monoaminergic modulation. There motoneuron excitability is strongly increased in the course of fight or flight reactions by the OA analog norepinephrine (Heckman et al., 2003). It will be interesting to test whether invertebrate motoneurons are also direct cellular targets of aminergic modulation and whether OA and TA exert opposite effects on motoneuron membrane excitability. Please note that our study refers to the modulation of flight motor behavior, but it is well known from larger insects that walking is also under octopaminergic/tyraminergic control (Mentel et al., 2008; Rillich et al., 2013). Although flight and leg motoneuron dendrites cover spatially separate area of the thoracic motor neuropils in both larger insects (Ramirez and Pearson, 1988) and *Drosophila* (Baek and Mann, 2009; Brierley et al., 2012), Naz positive glial arbors and central arbors of OA/TA containing neurons are present in both neuropil areas. Therefore, from a sole anatomical point of view similar central actions of TA and OA are possible for the modulation of walking motor behavior.

Given that OA and TA modulate not only motor behavior, but also learning and memory (Burke et al., 2012; Waddell, 2013; Wu et al., 2013) as well as states of motivation, aggression and addiction (McClung and Hirsh, 1999; Scholz et al., 2005; Hoyer et al., 2008; Zhou et al., 2008), identification of the molecular mechanisms of degradation and the cellular sites of action are likely of broad interest.

## Author Contributions

SR and CD helped conceiveing the study, conducted experiments, analyzed data and helped writing the manuscript. BA was the driving force to conceive the study, contributed reagents and helped writing the manuscript.

## Conflict of Interest Statement

The authors declare that the research was conducted in the absence of any commercial or financial relationships that could be construed as a potential conflict of interest. The reviewer AB declared a shared affiliation, though no other collaboration, with one of the authors BA to the handling Editor, who ensured that the process nevertheless met the standards of a fair and objective review.
